# Testicular Diffuse Large B-Cell Lymphoma—Clinical, Molecular, and Immunological Features

**DOI:** 10.3390/cancers13164049

**Published:** 2021-08-11

**Authors:** Marjukka Pollari, Suvi-Katri Leivonen, Sirpa Leppä

**Affiliations:** 1 Research Program Unit, Faculty of Medicine, University of Helsinki, 00014 Helsinki, Finland; suvi-katri.leivonen@helsinki.fi (S.-K.L.); sirpa.leppa@helsinki.fi (S.L.); 2 Department of Oncology, Tays Cancer Center, Tampere University Hospital, 33521 Tampere, Finland; 3 Department of Oncology, Comprehensive Cancer Center, Helsinki University Hospital, 00029 Helsinki, Finland

**Keywords:** testicular diffuse large B-cell lymphoma, lymphoma immunology, tumor micro-environment

## Abstract

**Simple Summary:**

Testicular diffuse large B-cell lymphoma (T-DLBCL) is a rare and aggressive lymphoma entity that mainly affects elderly men. It has a high relapse rate with especially the relapses of the central nervous system associating with dismal outcome. T-DLBCL has a unique biology with distinct genetic characteristics and clinical presentation, and the increasing knowledge on the tumor microenvironment of T-DLBCL highlights the significance of the host immunity and immune escape in this rare lymphoma, presenting in an immune-privileged site of the testis. This review provides an update on the latest progress made in T-DLBCL research and summarizes the clinical perspectives in T-DLBCL.

**Abstract:**

Primary testicular lymphoma is a rare lymphoma entity, yet it is the most common testicular malignancy among elderly men. The majority of the cases represent non-germinal center B-cell-like (non-GCB) diffuse large B-cell lymphoma (DLBCL) with aggressive clinical behavior and a relatively high relapse rate. Due to the rareness of the disease, no randomized clinical trials have been conducted and the currently recognized standard of care is based on retrospective analyses and few phase II trials. During recent years, the tumor microenvironment (TME) and tumor-related immunity have been the focus of many tumor biology studies, and the emergence of targeted therapies and checkpoint inhibitors has significantly modulated the field of cancer therapies. Testicular DLBCL (T-DLBCL) is presented in an immune-privileged site of the testis, and the roles of NF-κB pathway signaling, 9p24.1 aberrations, and tumor-infiltrating immune cells, especially immune checkpoint expressing lymphocytes and macrophages, seem to be unique compared to other lymphoma entities. Preliminary data on the use of immune checkpoint inhibitors in the treatment of T-DLBCL are promising and more studies are ongoing.

## 1. Introduction

Diffuse large B-cell lymphoma (DLBCL) is the most common aggressive B-cell malignancy with a distribution of 30–58% of all lymphomas previously classified as Non-Hodgkin lymphomas (NHLs) and has a consistently rising incidence. Depending on the clinical and biological risk factors, 50–90% of DLBCL patients can be cured with standard treatment options.

Extranodal presentation is a known clinical risk factor in DLBCL, and testicular DLBCL (T-DLBCL) is a rare and aggressive entity. T-DLBCL accounts for 1–2% of NHLs, with an incidence of 0.09–0.26/100,000 population, representing about 5% of all testicular malignancies. However, it is the most common testicular neoplasm in elderly men with a reported median age at diagnosis varying approximately between 60 and 70 years [1,2,3,4,5,6,7].

T-DLBCL arises primarily in the immune-privileged site of the testis and represents the most commonly activated B-cell-like (ABC) or non-germinal center B-cell-like (non-GCB) phenotype [6,7,8,9,10,11,12,13,14,15]. It has a high tendency of relapsing in other extranodal sites, typically the contralateral testis and the central nervous system (CNS), and especially relapses of the CNS, associated with poor prognosis [16].

Treatment of T-DLBCL is designated to gain both local and systemic control of the disease, as well as to prevent a possible relapse in the contralateral testis and the CNS. Due to the rareness of the disease, no randomized phase III trials have been conducted and the internationally recognized standard of care is based on data from phase II trials and retrospective analysis [1,17,18,19,20,21,22]. According to the few phase II trials, the median 5-year overall survival (OS) and progression-free survival (PFS) rates are 66–85% and 70–75%, respectively [18,19].

The tumor microenvironment (TME) of T-DLBCL has been demonstrated to harbor characteristic features that highlight the role of immune escape [23,24]. The TME consists of different host immune cells, stromal cells, blood vessels, cell matrices, as well as different cytokines, chemokines, and exosomes, and the role of host immunity in tumor evolution was originally recognized decades ago [25]. However, only recent advancements in the understanding of the complexity of the host-related factors and immunosurveillance have led to major achievements in the medical field, as checkpoint inhibitors and other immune modulators have shown their effectiveness in the treatment of several malignancies including lymphomas [25,26,27]. Although preliminary data on the use of immune checkpoint inhibitors seem promising in T-DLBCL, the understanding of the TME of the disease is only beginning to unfold [28].

We conducted a PubMed search to identify preclinical and clinical data on T-DLBCL and its TME. As the majority (75–90%) of primary testicular lymphoma cases represent DLBCL, our review focuses on T-DLBCL, leaving out other rare lymphoma entities with reported testicular involvement, such as testicular follicular lymphoma, mantle cell lymphoma, and Burkitt lymphoma [7].

## 2. Etiology and Pathogenesis

T-DLBCL is a malignancy of the B-cells, in which normal development and differentiation of B-cells are disturbed. Distinct mechanisms of lymphoma pathogenesis in the unique immune-privileged environment of the testis remain unsolved, but the majority of T-DLBCLs can be associated with the plasmablast/post germinal center (GC) phase of B-cell differentiation [29,30].

The original DLBCL cell-of-origin (COO) classification was based on gene expression profiling (GEP) of 3186 genes with known importance to lymphocyte and/or cancer biology and separated two distinct DLBCL entities: Tumors with germinal center B-cell-like (GCB) phenotype expressed genes characteristic to B-cells of the GC “light zone” and are associated with better prognosis in response to CHOP (doxorubicin-cyclophosphamide-vincristine-prednisone)-like chemotherapy, whereas tumors with ABC phenotype are associated with worse outcome and expressed genes that are induced during in vitro activation of peripheral B-lymphocytes, indicating that the tumor cells had experienced the GC phase and were closest to plasmablast stage of B-cell development [29]. As GEP is not available for routine clinical practice in all laboratories, more feasible techniques such as immunohistochemistry (IHC)-based algorithms have been applied in determining the COO [31,32,33,34]. The most commonly used IHC-based algorithm is the Hans algorithm that classifies COO into GCB or non-GCB phenotypes based on the expression of CD10, Bcl-6, and MUM1, and reported longer survival among patients with GCB phenotype even though later reports on the prognostic value of COO have been inconsistent [31,35,36,37,38,39,40]. Among T-DLBCLs, 60–96% of the cases have been reported to represent ABC/non-GCB phenotype, mainly based on Hans algorithm, although characterizing extranodal entities with IHC-based algorithms originally designed to classify nodal DLBCL is not unequivocally approved [6,7,9,10,11,12,41]. Only one of the studies used GEP, and in this study, the proportion of ABC phenotype was 96% [41].

Little is known about the etiology of T-DLBCL. HIV infection is a recognized risk factor for aggressive and primary extranodal lymphomas, and the only well-described etiological factor of T-DLBCL. Even though T-DLBCL mainly affects elderly men, in non-immune competent individuals such as patients with HIV/AIDS, it may also arise in younger patients [42,43].

The genetic aberrations in T-DLBCL, leading to oncogenic signaling, NF-κB pathway activation, and immune-escape phenotype, as well as characteristics of the TME, seem to distinguish T-DLBCL from nodal DLBCL, potentially highlighting the unique pathogenesis of T-DLBCL [12,23,44]. Additionally, mechanisms that protect the developing gametocytes in the immune-privileged site of the testis may serve T-DLBCL an exquisite milieu ideal for developing an immune-escape phenotype similar to PCNSL [13,15,45,46,47]. Besides the mechanical and functional properties of the blood barriers protecting these vulnerable organs, the definitive underlying mechanisms of the pathogenesis and evolution of these aggressive lymphomas remain unsolved.

## 3. Lymphoma Classification

Lymphoma entities are classified according to the World Health Organization (WHO) “Classification of Tumours of Haematopoietic and Lymphoid Tissues” that subdivides DLBCL into morphological variants, molecular subtypes, and clinically distinct disease entities [48].

T-DLBCL, as DLBCL in general, is defined as a lymphoma of medium or large B-cells that have a nuclear size equal to or exceeding normal macrophage nuclei or more than twice the size of a normal lymphocyte, with a diffuse growth pattern [46]. The neoplastic cells typically express pan-B-cell markers such as CD19, CD20, CD22, CD79a, and PAX5. Surface and cytoplasmic immunoglobulin (Ig), most commonly IgM, is demonstrated in the majority of the cases, and proliferation index Ki-67 is high [48].

Characteristic for T-DLBCL is its location in the immune-privileged site of the testis, where the blood–testis barrier protects the vulnerable testicular tissue from, e.g., harmful chemicals. Another aggressive lymphoma entity arising in an immune-privileged site is primary central nervous system lymphoma (PCNSL), with a similar blood–brain barrier protecting the brain. However, even though primary DLBCL of the CNS shares several biological features with T-DLBCL and is classified as its own entity by the WHO classification, T-DLBCL has not yet been recognized as a distinct DLBCL entity.

## 4. Clinical Presentation; Diagnosis, Staging, and Prognostic Factors

T-DLBCL typically presents as a firm, 4–6 cm sized, painless mass in the testis with no preference for either side. It is the most common bilateral testicular malignancy with synchronous bilateral involvement described in 6–15% of the cases [5,6,21,49]. T-DLBCL has a high relapse rate and the recurrence most often occurs in the CNS or the contralateral testis [4,6,16,21]. The involvement/recurrence of the CNS is especially associated with a dismal prognosis, and relapses in the CNS have been reported in up to 30% of the cases within 1–2 years from diagnosis, but relapses can also occur in other extranodal sites and up to 10 years after primary diagnosis [5,6,16,21]. B-symptoms (fever, loss of weight, and/or sweating) are rare and occur mainly in the case of disseminated disease, in 20–30% of the patients at diagnosis [5,6,16].

Histopathological diagnosis of T-DLBCL is obtained from a tissue sample of the involved testis. Staging is commonly classified by the Ann Arbor lymphoma staging with few modifications, and requires an ultrasound of the contralateral testis and contrast-enhanced computer tomography (CT) of the whole body and neck, with additional magnetic resonance imaging (MRI) of the brain and cytological and flow cytometric analysis of the cerebrospinal fluid recommended (Table 1) [22,50,51]. Whole-body 18-fluorodeoxyglucose positron emission tomography-computed tomography (18-FDG-PET-CT) is more sensitive in detecting possible other extranodal lymphoma lesions and is considered to be a part of the standard practice for both staging and response assessment [22,50,52]. Bone marrow biopsy is needed to assess possible lymphoma involvement but can be omitted if PET-CT-scan demonstrates bone disease [50]. The majority of T-DLBCL cases have limited stage disease with lymphoma only in the testis (stage IE). Approximately 20% have locally advanced stage II disease, whereas disseminated stage IV disease is virtually indistinguishable from a nodal DLBCL with testicular involvement.

Some clinical features, such as higher age at diagnosis and advanced stage (stage III–IV) disease, are well-recognized risk factors in DLBCL, and the same clinical factors are prognostic also in T-DLBCL [1,4,5,6,9,21,49,53,54,55,56,57,58]. However, at the moment, no specific prognostic score has been developed for T-DLBCL, although some clinical features such as absolute lymphocyte counts in the peripheral blood are shown to be predictive [59]. For the time being, the outcome of T-DLBCL patients can be estimated according to the International Prognostic Index (IPI) or the modified age-adjusted IPI (aaIPI), revised IPI (R-IPI), or the National Comprehensive Cancer Network IPI (NCCN-IPI) Table 2 and Table 3 [53,60,61,62].

## 5. Treatment

Besides reaching a complete remission, the aim of T-DLBCL treatment is to prevent relapses of the contralateral testis and the CNS. Due to the lack of randomized phase III trials, the treatment recommendations rely on two prospective phase II trials and retrospective data with consistent evidence on anthracycline-based chemotherapy improving the outcome of patients with T-DLBCL [17,18,19,21]. Although commonly included in the treatment regimen, the effect of anti-CD20 monoclonal antibody (mAb) rituximab on the survival and the risk of CNS relapse has not been thoroughly established and seems to be controversial, though retrospective data suggest it is beneficial, especially among high-risk patients [1,5,19,20,58,63]. Treatment of the contralateral testis, either surgical or irradiation, has been shown to reduce the risk of recurrence of the contralateral testis [1,18,19,57,64].

The optimal administration of CNS-targeted therapy has not been univocally defined. Results from a large Nordic retrospective analysis showed that systemic intravenously (IV) administered CNS-targeted chemotherapy correlates with a significantly improved overall survival in comparison to immunochemotherapy treatment with R-CHOP (rituximab-doxorubicin-cyclophosphamide-vincristine-prednisone)-like regimen alone, also among elderly (≥70 years at diagnosis) patients, with no difference in the CNS relapse rate, highlighting the role of systemic CNS-targeted therapy in gaining better systemic control in T-DLBCL [1]. However, no solid evidence on the benefit of intrathecal (IT) CNS-targeted chemotherapy has been shown even though some retrospective series from the pre-rituximab era reported improved PFS rates among patients treated with IT CNS-targeted chemotherapy [1,21,58].

Based on these data, the internationally recognized standard of care consists of orchiectomy followed by immunochemotherapy with six cycles of R-CHOP or R-CHOP-like regimen given every 21 days [22,50]. The addition of CNS prophylaxis with IV administered CNS-penetrating chemotherapy such as high dose methotrexate (HD-Mtx) or high dose cytarabine (HD-Ara-C) and/or IT chemotherapy as well as irradiation or excision of the contralateral testis are highly recommended [1,17,18,19,21,22,50].

## 6. Genetic Landscape

The genetic landscape of T-DLBCL shares similarities with nodal DLBCL, but also has its own unique characteristics. As the majority of T-DLBCLs represent the ABC/non-GCB phenotype, the frequency of *BCL2* and *MYC* rearrangements is comparable to nodal ABC-DLBCL (about 10% and 15%, respectively), while *BCL6* rearrangements seem less common in T-DLBCL (about 40% and 60%, respectively) [44]. Co-translocation (so-called double-/triple-hit lymphomas) and co-expression (so-called double-/triple-expressers) of these genes are associated with significantly worse outcomes as well as increased risk of extranodal involvement and CNS relapse in DLBCL in general, but the frequency of double-expressers has been reported to be markedly lower in T-DLBCL (about 13% in T-DLBCL and 20–30% in DLBCL in general) [12,38,65,66,67,68,69,70,71,72,73,74,75,76,77,78,79,80,81,82,83].

**Figure 1:**
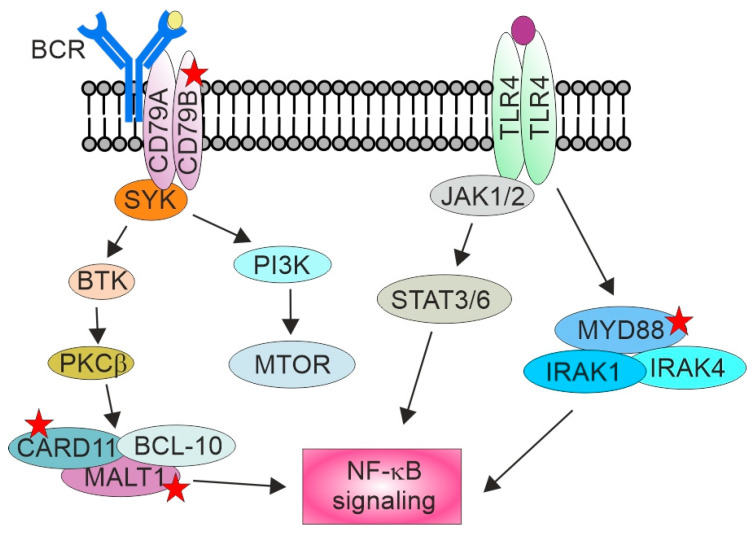
Dysregulated NF-κB signaling in T-DLBCL. Genomic alterations in the BCR and TLR signaling result in constitutive activation of NF-κB signaling. Aberrations typical for T-DLBCL are marked with red stars. BTK, Bruton’s tyrosine kinase; IRAK, interleukin-1 receptor-associated kinases; JAK, Janus kinase; MALT1, mucosa-associated lymphoid tissue; MTOR, mammalian target of rapamycin; PI3K, phosphoinositide 3-kinase; PKCβ, protein kinase C beta; STAT, signal transducer and activator of transcription; SYK: spleen-associated tyrosine kinase; TLR, toll-like receptor. Adapted from King et al., 2021 [88].

In more recent genetic studies, different stromal, host immune response, and lymphoma driver gene signatures have been shown to separate DLBCL patients into distinct subgroups with different genetic aberrations, phenotypes, and treatment responses [37,84,85,86,87]. The majority of T-DLBCL cases have been found to cluster among DLBCLs with worse prognosis and genetic alterations previously associated with ABC-DLBCLs, such as frequent mutations of *CD79B* and *MYD88* [85,86]. With additional near-uniform 18q gain, likely associated with increased expression of *BCL2* and other candidate drivers such as *MALT1*, these aberrations lead to oncogenic signaling and constitutive activation of the NF-κB pathway, ultimately resulting in increased cell survival and proliferation (Figure 1).

However, increasing evidence shows marked differences in the biology of T-DLBCL and nodal ABC-DLBCLs, with many of the unique genetic and molecular features of T-DLBCL shared with PCNSL, and summarized in Table 4 [8,9,12,23,45,46,47,89,90,91,92,93,94,95]. In T-DLBCL, rearrangements of *CD274* and *PDCD1LG2* (coding programmed cell death ligands 1 and 2, PD-L1 and PD-L2, respectively) and *BCL6* have been associated with increased risk of CNS relapse, and mutations of *TBL1XR1* and overexpression of p53 with inferior outcome [96,97,98]. Frequent copy number alterations and translocations of *9p24.1* resulting in increased expression of PD-L1/L2 proteins as well as loss of *HLA* genes are among the characteristic genetic features of T-DLBCL and PCNSL, highlighting the significant role of the TME and immune-escape in these diseases [8,9,95,99,100].

## 7. The Tumor Microenvironment

The role of host immunity and immune escape is becoming increasingly recognized in many cancers, including lymphomas [110,111,112]. Lymphomas can, however, be seen as a dysfunction of the immune system per se, as they develop from and are malignancies of the immune cells: B-cells, T-cells, or natural killer (NK) cells. The additional changes in the TME lead to a delicate and complex system with numerous different immune cells and pathways involved in the tumor pathogenesis and tumor evolution.

Tumors seem to have the capability of reprogramming host immune cells towards immunosuppressive activity. Reduced immunogenicity and escape from T-cell-mediated anti-tumor immune response can be caused by several mechanisms: direct interactions between the tumor and the host immune cells, altered expression of surface molecules leading to decreased recognition by the host immune cells or a state of T-cell exhaustion and impaired T-cell-mediated cytotoxicity, or recruitment of immunosuppressive cells that downregulate T-cell activation [113]. In T-DLBCL, the tumor cells can furthermore have additional mechanisms that can, e.g., enable their escape from the macrophage-mediated immune response.

The unique location of T-DLBCL and PCNSL in the immune-privileged sites of the testis and the CNS, protected by the blood–testis barrier and the blood–brain barrier, creates these malignancies a naturally distinct TME to develop in. The blood–testis barrier consists of three components that suppress detrimental immune responses against auto-antigenic germ cells [14]. The anatomical barrier restricts the passage of molecules and cells, the physiological barrier regulates the movement of substance, and the immunological barrier limits access by immunological mechanisms. Together these barriers create a microenvironment ideal for the proper development and maturation of germ cells as well as unique for lymphoma pathogenesis and evolution. The increasing knowledge on the characteristics of the TME of T-DLBCL, and the prognostic role of certain host immune cells widens the understanding of the biology of the disease, with hopefully also applications to therapeutic approaches in the future.

### 7.1. Tumor-Infiltrating Lymphocytes

Tumor-infiltrating lymphocytes (TILs) are a heterogeneous and versatile group of immune cells that have a dominant role in developing a host immune response against tumor cells [114,115,116]. CD8^+^ cytotoxic T-lymphocytes (CTLs) are essential in cell-mediated immune defense while regulatory T-cells (Tregs) reduce the responses of both innate and adaptive immune systems and collaborate with numerous different immune cell subtypes including M2 macrophages and cancer-associated fibroblasts (CAFs) [117]. T helper (Th) cells differentiate from naive CD4^+^ T-cells, are needed in adaptive immune response, and provide help to both B-cells and T-cells as well as assist in activating and promoting DC maturation and function [118]. Th cells can be further divided into subgroups such as Th1 cells that are essential in the cell-mediated immune response against intracellular pathogens and have a crucial role in effective anti-tumor immunity, and Th2 cells that drive host defense against extracellular parasites and suppress the differentiation of Th1 cells and function of DCs [118]. In the TME, Th cells can also promote the priming, activity, and effector and memory functions of CTLs [119].

**Figure 2:**
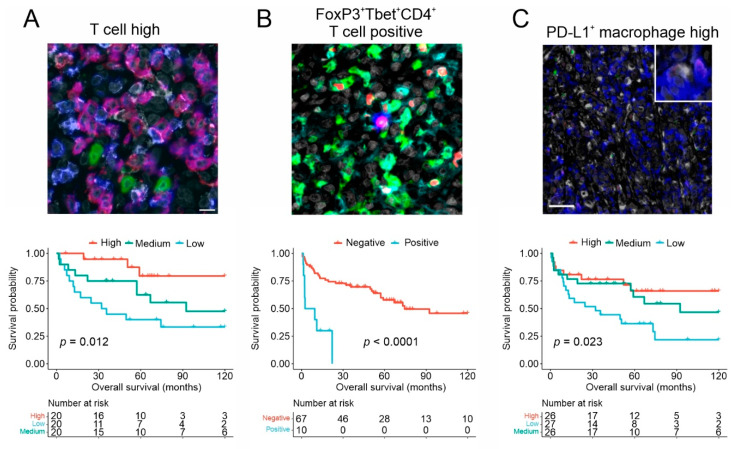
Examples of distinct cell types in the T-DLBCL and their impact on survival. (**A**) Multiplex-IHC (mIHC) image (upper panel) showing high proportion of T cells in T-DLBCL TME. CD3 = blue, CD4 = white, CD8 = red, and CD56 = green (scale bar 20 μm). In the lower panel, Kaplan Meier plot demonstrating the impact of high T cell proportions on improved survival in T-DLBCL patients. (**B**) In the upper panel mIHC image showing FoxP3^+^Tbet^+^CD4^+^ T cells in the T-DLBCL TME. CD3 = green, CD4 = cyan, FoxP3 = red, T-bet = blue. In the lower panel Kaplan Meier plot demonstrating the inferior effect of FoXP3-Tbet-positivity on T-DLBCL survival. (**C**) In the upper panel, mIHC image showing high levels of PD-L1-positive macrophages in the T-DLBCL TME. PD-L1 = blue, PD-L2 = red, CD68 = white, c-Maf = green (scale bar 40 μm). In the lower panel, Kaplan–Meier plot demonstrating the impact of low PD-L1-positive macrophages on poorer survival in T-DLBCL. The figure was reproduced and adapted from Pollari et al., 2018, Leivonen et al., 2019, and Pollari et al., 2020 with the permission of the Ferrata Storti Foundation (**A**,**C**) and John Wiley and Sons, Inc. (Hoboken, NJ, USA) (**B**) [23,24,120].

In T-DLBCL, higher content of CD3^+^ TILs in general (Figure 2A), as well as higher proportions of both CTLs and CD4^+^ TILs, has been demonstrated to be associated with favorable outcomes [23]. Higher expression of a 121-gene T-lymphocyte signature was demonstrated to correlate with longer survival, especially among rituximab-treated T-DLBCL patients, and high expression of individual T-lymphocyte surface markers such as *CD3D*/*E*/*G*, *CD4*, and *CD8A*/*B* was shown to translate to favorable outcomes, emphasizing the prognostic impact of TILs [23]. Lower expression of *HLA* class I and II genes, that code MHC class I and II proteins needed to initiate the cellular immune response by presenting antigens to T-cells, and their membrane expression was shown to be associated with low expression of the T-lymphocyte signature genes and worse prognosis [23].

The function of TILs is highly complicated, and although beneficial in infections, uncontrolled Th1 response against self-antigens can, for example, lead to an aggressive autoimmune disease [121]. Therefore, several distinct mechanisms of the immune system and the TME regulate the activity of TILs. In addition to the self-regulatory mechanism of highly activated TILs, other immune cells, such as CAFs, M2-like macrophages, and Tregs, can downregulate the activity of TILs, with Tregs having a significant role in regulating both Th1 cells as well as CTL-mediated anti-tumor immune response [122,123,124]. During a strong Th1 response, Tregs have been demonstrated to be able to differentiate into a subgroup of Tregs that express both forkhead box P3 (FoxP3) and T-box transcription factor TBX21 (T-bet), which are optimized to suppress the activity of Th1 cells, and are associated with tumor growth [114,115,121,125,126,127,128,129]. Additionally, in normal conditions, the expression of inhibitory receptors such as programmed cell death-1 (PD-1) and cytotoxic T-lymphocyte-associated protein 4 (CTLA-4) on T-cells downregulate the excessive immune response, autoimmune activity, and tissue damage. In the TME, the continuous antigen exposure can, however, drive CTLs into a state of exhaustion with decreased cytokine production, reduced proliferation and cytotoxic activity, and induced expression of the inhibitory receptors [110,130,131,132]. In T-DLBCL, higher proportions of PD-1 expressing CTLs and CD4^+^ TILs have been shown to correlate with significantly longer survival while a subpopulation of FoxP3^+^T-bet^+^ double-positive Tregs has been identified to have a significant adverse effect on outcome (Figure 2B) [23,24,120]. Together with the preliminary promising results with PD-1 targeting mAbs in T-DLBCL, these findings highlight the exquisitely delicate TME of the disease, although leave more detailed questions about the function of these immune cells unresolved [23,24,28].

### 7.2. Macrophages

Macrophages have a role in both innate and adaptive immune systems and can derive into different subtypes with distinct functions according to their microenvironment [133,134]. Tumor-associated macrophages (TAMs) have been demonstrated to have alternative roles depending on the tumor phase and other TME-related factors, and seem to be able to convert from tumor suppressive M1-like macrophages into pro-tumor M2-like macrophages and vice versa [133,135]. Especially in the early stages of tumor evolution, TAMs have been described to secrete pro-inflammatory cytokines and have M1-like tumor-suppressive functions such as elimination of the tumor cells, and the immunosuppressive phenotype of TAMs has been associated with defective activation of NF-κB [136,137]. The phagocytotic activity of TAMs can be inhibited by factors such as NF-κB-regulated expression of immune checkpoint molecules like PD-1, and TAMs have also been reported to resemble M2 macrophages with a decreased ability to lyse tumor cells and a capability of directly promoting tumor angiogenesis, tumor growth, and metastasis [136,138,139,140,141].

In T-DLBCL, a marked proportion of TAMs and lymphoma cells have been shown to express PD-L1, and higher proportions of PD-L1^+^CD68^+^ TAMs have been associated with longer survival (Figure 2C) [24,142]. Unlike in DLBCL in general, no association to survival could be seen with the overall proportion of CD68^+^ TAMs in T-DLBCL, and the proportion of PD-L1^+^CD68^−^ cells did not seem to have an effect on the outcome [24,143,144]. Instead, higher overall expression of PD-L1 was demonstrated to translate to superior outcomes, and although the association seems to be especially related to the expression on CD68^+^ TAMs, a higher content of PD-L1^+^ lymphoma cells may be advantageous, though this finding has not been consistent in smaller studies [24,142].

TAMs have several different mechanisms for regulating the activity of T-cells [145]. As one of the main functions of macrophages is to induce T-cell recruitment and activation, in the TME, this can be disturbed by the poor tumor-associated antigen-presenting capability of TAMs. Additionally, M2-like TAMs can decrease T-cell activation by suppressing the function of other APCs such as DCs, shift the balance of Th1/Th2 differentiation towards Th2 cell activation, and induce the activation of Tregs, leading to further suppression of immune response against the tumor. In T-DLBCL, the content of PD-L1^+^CD68^+^ TAMs has been shown to correlate with the content of PD-1^+^CD4^+^ TILs and PD-1^+^ CTLs, suggesting that these TAMs with a favorable prognostic value might more likely represent an M1- than M2-like phenotype [24]. The finding that PD-L1^+^CD163^+^ TAMs, denoting M2-like phenotype, was not associated with survival supports the assumption that the favorable prognostic impact of TAMs is presumably associated with PD-L1 expressing M1-like phenotype in T-DLBCL [24].

### 7.3. The Role of Host Immunity and Immune-Escape

The role of host immunity and immune-escape seem to have special importance in T-DLBCL. The distribution of TILs and TAMs, as well as their expression of immune checkpoint molecules, has been shown to have a great variation and is associated with survival [24,120]. Copy number losses of *HLA* class I and II genes resulting in decreased expression of MHC I and II and limited tumor-antigen presentation to TILs, gains and amplifications of *CD274* and *PDCD1LG2* with their increased transcription and PD-L1/PD-L2 protein expression implicating immune escape, as well as T-DLBCL’s location in an immune-privilege site are all hallmarks of T-DLBCL and highlight the significance of the TME and host-related factors [45,47,99,146]. Higher proportions of PD-L1^+^CD68^+^ TAMs correlating with higher content of PD-1^+^CD4^+^ TILs and PD-1^+^ CTLs and a favorable prognosis further emphasize the complexity of the PD-1–PD-L1 pathway and its role in host immunity [24]. The content of other immune cells of the TME, such as NK cells, seems to be markedly low, and their possible role in T-DLBCL remains to be further studied [23].

The TME has an important role in achieving treatment response. Both CD4^+^ T-cells and CTLs as well as complement activation, NK cells, neutrophils, and macrophages have been reported to be involved in the Ab-dependent cell-mediated cytotoxicity [129,147,148,149,150,151,152,153,154,155,156]. In T-DLBCL, the overall beneficial role of TILs and PD-L1^+^ TAMs was shown to be especially evident among immunochemotherapy-treated patients and can therefore relate to the crucial role of host immunity and the pre-existing TIL and TAM populations that can induce a response to both chemotherapy agents and rituximab. Additionally, as HLA proteins are crucial in antigen presentation and tumor cell recognition, loss of HLA protein expression may, in addition to facilitating lymphoma cells in their escape from immunosurveillance, also lead to impaired recruitment of TILs and inadequate response to therapeutic agents, which is in line with the association of *HLA* class I and II and the T-lymphocyte signature gene expression levels and worse prognosis particularly in rituximab-treated T-DLBCL patients [23,46,47,90,94,100].

Increasing knowledge on the complexity of the TME and host-related factors involved in the tumor immune-escape is continuously revealing new therapeutic opportunities [25,27]. Immunotherapy and especially host T-cell-specific immune response promoting therapies have lately been the main focuses of clinical trials in many malignancies including lymphomas [119,124,157,158,159]. Taking into account the reports on PD-L1 expression on tumor cells associated with immune-escape and T-cell non-responsiveness, findings on PD-1 expression on CTLs, as well as promising preliminary results with PD-1 blockade therapy in T-DLBCL, the results on immune checkpoint molecule expression in T-DLBCL can be seen somewhat paradoxical [28]. The PD-1–PD-L1 pathway and its functions in the TME have, however, been shown to be much more complicated. Besides PD-1, also additional binding sites to PD-L1 and PD-L2 have been described [160,161,162]. Co-expression of immune checkpoint molecules including PD-1 have been demonstrated to define a group of not merely exhausted, but highly activated and functional CTLs [163]. PD-1 expression on TAMs has been associated with inhibited phagocytosis and tumor immunity, and blockade of the PD-1–PD-L1 pathway can reverse this [140]. Conversely, blockade of PD-1 was demonstrated to increase the proliferation and activity of antitumor immune response suppressive Tregs [164].

As a conclusion, the role of the TME and the favorable prognostic value of immune checkpoint expressing TILs and TAMs is especially evident in T-DLBCL, whereas no such positive association with survival has been observed in nodal DLBCL [23,24,165]. In contrast, a higher proportion of TILs with immune checkpoint expression has been demonstrated to translate to unfavorable survival among DLBCL patients in general [165,166]. However, PD-1 blockade with nivolumab has shown only modest responses in patients with relapsed/refractory DLBCL in general [167]. While the role of immune checkpoint inhibitors and other targeted therapies in T-DLBCL remains to be established, preliminary results have been encouraging and a phase II study of nivolumab in relapsed and refractory PCNSL and T-DLBCL patients (NCT02857426) is currently ongoing.

## 8. Conclusions and Future Perspectives

T-DLBCL is a unique entity of aggressive B-cell lymphomas with a characteristic genetic profile that highlights significant NF-κB/TLR-mediated signaling, often with concurrent B-cell receptor pathway activation. The distinct predictive value of the TME together with the current knowledge on the PD-1-PD-L1 signaling and suggested interactions between tumor cells and the host immune cells highlights the clinical relevance of host-related factors in the TME of T-DLBCL. As the overall beneficial roles of TILs and PD-L1^+^ TAMs seem especially evident among immunochemotherapy-treated T-DLBCL patients, the data suggest that the TME and the pre-existing TIL and TAM populations have a crucial role in inducing response to both chemotherapeutic agents and rituximab. However, the adverse effect of FoxP3^+^T-bet^+^ double positive Tregs remains to be further defined, and future studies are awaited to better characterize the cell-to-cell interactions and spatial heterogeneity of the TME as well as to determine the optimal targets for immunotherapies. Results from ongoing phase II studies and future clinical trials will determine the role of PD-1–PD-L1 blockade, and the increasing understanding of the TME will hopefully bring more treatment options, also with commonly well-tolerated mAbs, in treating patients with T-DLBCL in the future. Targeting the adverse prognostic factors of the TME could bring significant benefit to patients that do not respond to the current standard of care.

## Figures and Tables

**Table 1:** Ann Arbor lymphoma stage classification. In T-DLBCL, stage III-IV is defined as advanced stage with mono or bilateral testicular involvement with involvement of distant lymph nodes and/or extranodal sites.

Stage	Description
I	Involvement of a single lymphatic region (I) or localized involvement of single extranodal organ or site (testis mono or bilateral) (IE)
II	Involvement of two or more lymphatic regions on the same side of the diaphragm (II) or localized involvement of a single extranodal organ or site (testis mono or bilateral) and of one or more lymphatic regions on the same side of the diaphragm (locoregional lymph nodes; iliac and/or lomboaortic) (IIE)
III	Involvement of lymphatic regions on both sides of the diaphragm
IV	Diffuse or disseminated involvement of one or more extranodal organs with or without lymphatic involvement

**Table 2:** International Prognostic Index. 1 score for each: Stage III–IV, elevated Lactate Dehydrogenase, age over 60 years at diagnosis, ECOG performance status 3–4, more than 1 extranodal lymphoma site.

Risk Group	IPI Score	3-Year OS, (95% CI), %
Low	0 or 1	91 (89–94)
Intermediate low	2	81 (76–86)
Intermediate high	3	65 (58–73)
High	4 or 5	59 (49–69)

**Table 3:** Age-adjusted International Prognostic Index (aaIPI). 1 score for each: Stage III–IV, elevated Lactate Dehydrogenase, ECOG performance status 3–4.

Risk Group	aaIPI Score	3-Year OS, (95% CI), %
Low	0	98 (96–100)
Intermediate low	1	92 (87–95)
Intermediate high	2	75 (66–82)
High	3	

**Table 4:** Genetic alterations characteristic of T-DLBCL.

Gene/Chromosome	Aberration	Occurrence in T-DLBCL	Occurrence in DLBCL	Occurrence in Non-GCB-DLBCL	Ref.
*NFKBIZ*	Copy number gain	42%	9%	20%	[45]
*MYD88*	Amplifications, mutations, and deletions	60–82%	18–27%	29%	[8,45,91,92,101]
*CD79b*	Mutations and deletions	19–34%	14–15%	23%	[91,92,101,102]
*CDKN2A*	Copy number alterations	71%	24%	35%	[45]
9p24.1	Translocation and copy number alterations	54%	<10%	<10%	[45]
*CD274*	Rearrangements, copy number alterations, and increased protein expression	35%	27%	45%	[45,89,103]
*PDCD1LG2*	Rearrangements, copy number alterations, and increased protein expression	47%	<5%	ND ^1^	[45,89,104]
*pSTAT1/pSTAT3*	Expression	82%	ND	ND	[12]
*BCL2/MYC*	Rearrangements	10–15%	10–30%	~35%	[44,101,105]
*BCL6*	Rearrangements and deregulation	16–48%	35%	19–60%	[12,45,86,91,106,107]
*CIITA*	Rearrangements	10%	3–6%	ND	[89,101]
*FOXP1*	Rearrangements and increased protein expression	7–78%	8–10%	15–30%	[9,89,106,108]
*HLA* region/genes	Mutations, deletions, and loss of expression	61–77%	4–22%	ND	[23,46,47,90,94,95,101]
IgH	V(D)J rearrangement and SHMs	43%	<80%	ND	[9,109]
*CD37*	Mutations	26%	ND	ND	[93]

^1^ ND, not determined.
